# The nuclear receptor gene family in the Pacific oyster, *Crassostrea gigas*, contains a novel subfamily group

**DOI:** 10.1186/1471-2164-15-369

**Published:** 2014-05-15

**Authors:** Susanne Vogeler, Tamara S Galloway, Brett P Lyons, Tim P Bean

**Affiliations:** School of Biosciences, College of Life and Environmental Sciences, University of Exeter, Stocker Road, Exeter, EX4 4QD UK; Centre for Environment, Fisheries and Aquaculture Science, Cefas Weymouth Laboratory, Barrack Road, Weymouth, DT4 8UB UK

**Keywords:** Mollusc, Hormone receptor, Bivalve, Xenobiotics, Transcription factor

## Abstract

**Background:**

Nuclear receptors are a superfamily of transcription factors important in key biological, developmental and reproductive processes. Several of these receptors are ligand- activated and through their ability to bind endogenous and exogenous ligands, are potentially vulnerable to xenobiotics. Molluscs are key ecological species in defining aquatic and terrestrial habitats and are sensitive to xenobiotic compounds in the environment. However, the understanding of nuclear receptor presence, function and xenobiotic disruption in the phylum Mollusca is limited.

**Results:**

Here, forty-three nuclear receptor sequences were mined from the genome of the Pacific oyster, *Crassostrea gigas.* They include members of NR0-NR5 subfamilies, notably lacking any NR6 members. Phylogenetic analyses of the oyster nuclear receptors have been conducted showing the presence of a large novel subfamily group not previously reported, which is named NR1P. Homologues to all previous identified nuclear receptors in other mollusc species have also been determined including the putative heterodimer partner retinoid X receptor, estrogen receptor and estrogen related receptor.

**Conclusion:**

*C. gigas* contains a highly diverse set of nuclear receptors including a novel NR1 group, which provides important information on presence and evolution of this transcription factor superfamily in invertebrates. The Pacific oyster possesses two members of NR3, the sex steroid hormone receptor analogues, of which there are 9 in humans. This provides increasing evidence that steroid ligand specific expansion of this family is deuterostome specific. This new knowledge on divergence and emergence of nuclear receptors in *C. gigas* provides essential information for studying regulation of molluscan gene expression and the potential effects of xenobiotics.

**Electronic supplementary material:**

The online version of this article (doi:10.1186/1471-2164-15-369) contains supplementary material, which is available to authorized users.

## Background

### Nuclear receptors

**Figure 1:**
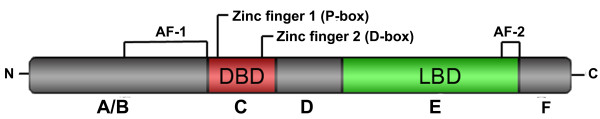
**Nuclear receptors gene structure.** The six regions **(A-F)** of nuclear receptors. The **A**/**B** region contains the AF-1 activation function. The highly conserved central DNA-binding-domain DBD (**C** region) comprises two zinc fingers, including the P-box and D-box. The ligand-binding-domain LBD (**E** region) contains the AF-2 activation function helix. Situated between the DBD and the LBD is the variable “hinge” region (**D** region). The C-terminal region **F** is located at the end of the NR and varies in length among different nuclear receptors.

Nuclear receptors (NRs) are transcription factors, which regulate the expression of specific genes involved in embryonic development, homeostasis and physiologically regulated processes. They are of particular interest as this regulation often requires interaction with endogenous or exogenous ligands. Nuclear receptors bind to a response element in target gene promoters and activate gene transcription in cooperation with bound co-factors [1]. Although activation of nuclear receptors often requires interaction with ligands, there are many proteins which can be ‘constitutively activated’ and perform their biological response without a ligand. Nuclear receptors are usually found in protein complexes as monomers, homodimers, or heterodimers [2], with one member of the NR2 family, the retinoid X receptor (RXR) operating as the predominant heterodimer partner in vertebrates [3]. Structures of nuclear receptors are well characterized and usually contain six common structural features (Figure 1). The A/B region and the final F region are highly variable and account for most of the difference observed between genes. The A/B region contains the activation function AF-1, which is able to synergize with AF-2 in region E to produce a more stable up-regulation of gene expression. Region C, the central specific DNA binding domain (DBD), is a highly conserved domain including two C_4_ zinc-fingers (alpha helices): (1) a five amino acid sequence (P-box) determining the specificity of DNA binding, and (2) the D-box, which mediates receptor dimerization. Region D is a flexible “hinge” domain and connects the DBD with region E, the ligand binding domain (LBD). The LBD is highly conserved in structure, and moderately conserved in sequence. It is often able to bind specific hormonal (e.g. thyroids, steroids) or non-hormonal (morphogens, dietary components) hydrophobic ligands and it can induce or inhibit the expression of a gene by a conformational change of the receptor [1].

Nuclear receptors are exclusive to multicellular metazoans. Their numbers in animals range from a few receptors in sponges and *Trichoplax*[4–7], to approximately 21 NRs in *Drosophila melanogaster*[8] and 48 NRs in humans. The nematode *Caenorhabditis elegans* possesses the highest number of NRs identified in a species with over 270 NRs [9]. Two nuclear receptors have been found in the demosponge *Amphimedon queenslandica*, suggesting that nuclear receptors originated from a single nuclear receptor in the ancestral metazoans [7]. This theory is supported by the deep conservation of the DBD and the LBD sequences between different animal phyla and suggests the divergence of nuclear receptors is most likely driven by gene duplication and gene loss [7, 10–12].

Nuclear receptors are divided into six subfamilies based on phylogenetic reconstructions of the DBD and LBD [13]. Abnormally structured NRs, which do not contain one of the two conserved regions (DBD or LBD), are grouped in a separate subfamily (NR0) irrespective of their phylogenetic relationship. A novel group of NRs has been identified in Platyhelminthes *Schistosoma mansoni,* containing tandem DBDs and a single LBD, which do not belong to the miscellaneous NR0 subfamily and are categorised as 2DBDNR group [14, 15].

### Nuclear receptors in the Mollusca

Mollusca (gastropods, bivalves, cephalopods and relatives) diverged rapidly during the Cambrian period resulting in a large range of morphology and life histories, becoming the second most species-rich phylum among the invertebrates [16]. Molluscs sit within the Lophotrochozoa, one of the two major groups among the protostomes. Marine mollusc species are common inhabitants of rocky, intertidal and estuary flats world-wide. They occupy important ecological niches as filter feeders and decomposers, and serve as a protein source for animals, including humans, linking them directly with human health. Molluscs are recommended as ideal sentinel species in a number of marine monitoring programmes including those supported by international bodies such as ICES and OSPAR [17]. Their large differences in anatomy and life cycle, their wide global distribution, bioaccumulation of chemicals by filtration and relatively straightforward capacity to be cultured and handled in the laboratory make molluscs an ideal species for studying biological processes. They are also often considered as surrogates for vertebrate models in laboratory based chemical risk assessment studies [18]. However, the information on similarities and differences between vertebrate and mollusc endocrine system and gene regulation is limited and a deeper insight as to how molluscs are affected by chemicals will therefore directly aid the development of ecological and chemical risk assessment and enhance protection of the marine environment.

Several NR sequences, including the conserved domains, have previously been isolated and characterized in molluscs. The estrogen receptor ER (NR3A) in the gastropod *Aplysia californica*[19] was the first NR identified in a mollusc species. Since then single ER homologs have been identified in more than eleven species among three main classes of the phylum Mollusca: gastropods (6), bivalve (4) and cephalopods (1) (Additional file 1). A second member of the NR3 subfamily, the estrogen related receptor, ERR, has been cloned in the gastropod snail *Marisa cornuarietis*[20] and a single RXR (NR2B) representative has been identified in at least six species among the molluscs (Additional file 1). Additionally, a retinoid acid receptor RAR (NR1B) has been cloned from the central nervous system of the pond snail *Lymnaea stagnalis*[21]. Finally, three molluscan receptors have been reported in the bivalve *Mytilus galloprovincialis,* which possesses one homolog to the NR1D group, one nuclear receptor closely related to the NR1D, NR1E and NR1F groups, and one receptor related to the nematode and trematode receptors SEX-1 (NR1G) [22].

### Nuclear receptors and xenobiotics

Due to their ligand binding abilities, some nuclear receptors are inherently vulnerable to xenobiotics, which can modulate normal gene expression by mimicking a ligand or blocking the LBD of nuclear receptors [23]. This can lead to abnormal gene expression and hence, to disruption of development and/or endocrinology of an organism. Various xenobiotics, which have a mode of action mediated through NRs, have thus been characterized as “endocrine disrupting chemicals” (EDCs). Published reports have interpreted EDCs as having caused serious effects of chemicals to the health conditions of human and wildlife [24, 25]. Mass mortalities and population declines in approximately 200 gastropod species worldwide [25–29] have been associated with exposure to tributyltin (TBT). This biocide was employed in antifouling paint on ships and fishing nets from the early 60s until 2005, when its use was legally restricted. In gastropods, exposure to TBT causes irreversible superimposition of male genital on females, a condition termed imposex, whilst in bivalve species, exposure to TBT causes growth reduction [30–32], and shell thickening [33–36]. The mechanism by which TBT affects mollusc species remains unclear, although hypotheses have been raised related to binding to and disruption of a putative molluscan RXR or RXR/peroxisome proliferator-activated receptor (PPAR) heterodimer [29, 37–47].

In this study, we took advantage of the recently released complete genome of the Pacific oyster *Crassostrea gigas*[48] to analyse the nuclear receptor gene family using a combination of bioinformatics and phylogenetics. Here we report the phylogenetic relationship of 43 NRs, confirm expression and discuss their homology to *Homo sapiens*, *D. melanogaster*, *C. elegans* and previously cloned molluscan NRs. The data are assessed from the perspective of putative function, evolution and the potential for the binding of xenobiotics, based on previous functional studies on nuclear receptor homologs in other species.

## Results

### Nuclear receptor genes

Forty-three putative NR genes were identified in the *C. gigas* genome. Transcription of all nuclear receptor genes was successfully confirmed by sequencing. Putative nuclear receptor affiliation was verified based upon the conserved domains, DBD and LBD, using a PFAM analysis and a conserved domain search resulting in 38 NRs showing the classical structures of the NR superfamily. One of the NR identified putatively appeared to suggest a sequencing error in the genome project, with a single LBD and a lack of DBD. Therefore, the full gene was re-sequenced as the NR homolog, CgNR1D, showing a single DBD and a single LBD. Five putative oyster NRs have abnormal structures including two NRs containing two DBDs and a single LBD, one NR lacking the DBD but containing a single LBD, and two NRs with only a single DBD and lack of a LBD. A full list of annotated protein sequences of *C. gigas* NRs including accession numbers is provided in the Additional file 1.

### Phylogenetic analysis

**Figure 2:**
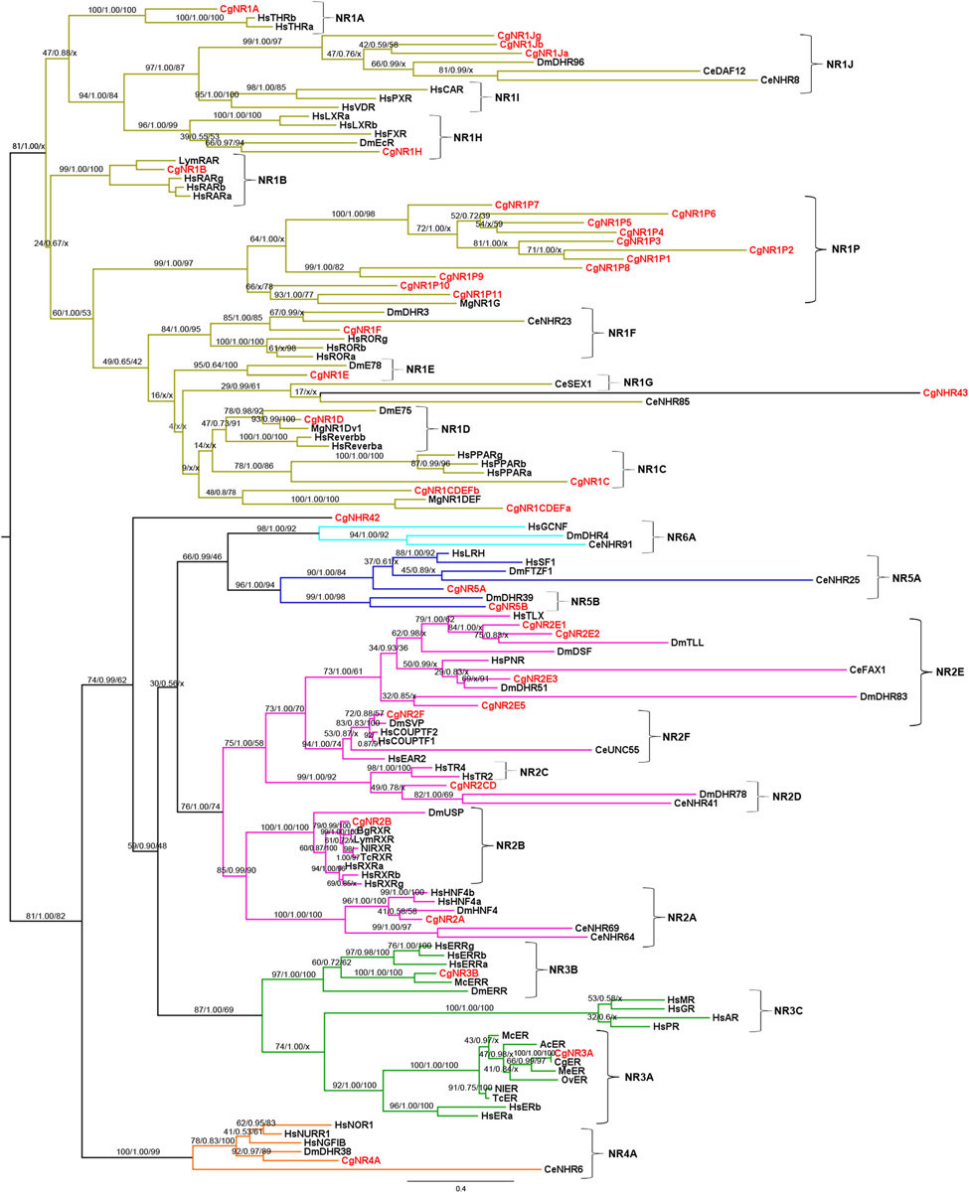
**Phylogenetic relationship of nuclear receptors in** ***Crassostrea gigas,*** ***Homo sapiens,*** ***Drosophila melanogaster,*** ***Caenorhabditis elegans*** **and mollusc species.** The alignment was constructed using the DBD plus portion of LBD and phylogenetic relationship was conducted by a Maximum likelihood (ML), Bayesian Inference and neighbour-joining (NJ) analyses. ML bootstrap support values (percentage of 1000 BS), Bayesian posterior probabilities (PPs) and NJ bootstrap support values (percentage of 1000 BS) are provided above the nodes separated by slash. Star indicates the node obtained from the Bayesian Inference and NJ analyses, which were different from that obtained by ML method. Highlighted clades display the six NR subfamilies, olive: NR1, pink: NR2, green: NR3, orange: NR4, dark blue: NR5, light blue: NR6. *C. gigas* NRs highlighted in red. Ac: *Aplysia californica,* Bg: *Biomphalaria glabrata,* Ce: *C. elegans*, Cg: *C. gigas,* Dm: *D. melanogaster*, Hs: *H. sapiens*, Lym: *Lymnaea stagnalis*, Mc: *Marisa cornuarietis*, Me: *Mytilus edulis*, Mg: *Mytilus galloprovincialis*, Nl: *Nucella lapillus,* Ov: *Octopus vulgaris,* Tc: *Thais clavigera.*

Phylogenetic analyses were performed using the amino acid sequences of the 43 *C. gigas* nuclear receptors. Several trees were constructed using different phylogenetic analyses: the DBD tree, based on just DNA binding domains (Maximum Likelihood (ML) and Bayesian Inference analyses, 38 classically and 4 abnormally structured *C. gigas* NRs); LBD tree, based on a portion of the LBD (ML and Bayesian Inference analyses, 38 classically and 3 abnormally structured *C. gigas* NRs); and DBD plus LBD trees, based on a composition of DBD and a portion of LBD (ML, Bayesian Inference and neighbor-joining (NJ) analyses, 38 classically structured *C. gigas* NRs). The ML and Bayesian Inference phylogenetic analyses of the DBD plus LBD alignment showed similar patterns and both segregated in a monophyletic group NR1 and a second major clade containing the subfamilies NR2-NR6 (Figure 2). The second major group further subdivided in five sub-clades NR2, NR4, NR3, NR5 and NR6. Nodes for NR1, NR3-NR6 were supported by high ML bootstrap scores (BS =81-100) and high posterior probabilities (PP = 0.99-1). The NR2 clade was more moderately supported (BS = 76), but highly supported by posterior probabilities (PP = 1). The NJ analysis of the DBD plus LBD segregated in three major clades: two including NR1 subfamily members and the third subdivided in the other NR2-NR6 subfamilies, which displayed different positioning of the NR3, NR5 and NR6 subfamilies compared to the ML and Bayesian Inference analyses. In general, the neighbor-joining analysis provided the lowest resolution of the DBD plus LBD trees and therefore, was only used as additional support for individual receptor placements. The individual ML and Bayesian Inference analyses of the separate DBD and LBD sequences resulted in less supported nodes for the six receptor subfamilies and were also not able to assign some of the receptor subfamilies to the existing monophyletic subfamilies (Additional files 2 and 3). Therefore, the phylogenetic relationship of the putative *C. gigas* nuclear receptors were deduced from the DBD plus LBD ML analysis supported by the Bayesian Inference analysis and NJ bootstraps values (Figure 2).

*C. gigas* possesses nuclear receptors belonging to six of the seven NR subfamilies. Twenty-three out of 38 classically structured oyster nuclear receptors are members of the NR1 subfamily (Figure 2). A novel NR1 group, NR1P, has been formed including eleven *C. gigas* receptors and the *M. galloprovincialis* nuclear receptor MgNR1G. Subfamily NR2 is represented by eight oyster NRs. There are also two NR3 members, one NR4 member and two NR5 members. No homologs to the NR6 receptor subfamily were identified in the *C. gigas* genome. The abnormally structured *C. gigas* receptor CgNR0B showed homologies to the miscellaneous subfamily NR0B (Additional file 3). CgNHR40 and CgNHR41, the two single DBD sequences, assigned to the CgNR1Ja receptor and outside the NR4 group, respectively (Additional file 2). The two nuclear receptors containing double DBDs and a single LBD display phylogenetic relationship to the 2DBD nuclear receptor group previously identified in *S. mansoni* (Additional files 2 and 3). Cg2DBDγ showed structural and amino acid identities to the Sm2DBDγ, with two DBDs and the LBD sequence identities of 55%, 60% and 25% respectively. Cg2DBDδ, however, does not display such a high relationship and its second DBD clusters outside the 2DBD receptor group close to CgNR1CDEFα and MgNR1DEF (ML analysis DBD). In addition, its LBD was weakly supported in the ML analysis and not supported by the Bayesian Inference analysis.

The large majority of *C. gigas* nuclear receptor assignments to subfamily groups were supported by high ML bootstrap scores (BS = 89-100) and Bayesian posterior probabilities (PP = 1). Exceptions include CgNR1C and the entire NR2E group containing four *C. gigas* NRs, which were highly supported by the Bayesian Inference analysis (both PP = 1) but only moderately supported by ML (BS = 78 and 73, respectively). Further positions within the groups among phylogenetic trees were fixed apart from the following exceptions. Two classically structured nuclear receptors (CgNHR42 and CgNHR43) could not be assigned to one of the receptor subfamilies. CgNHR42 was located as an outgroup to NR2/3/5/6 clade in the DBD plus LBD analyses and the LBD analyses, while the DBD analyses nested it inside the NR1 subfamily. BLASTp search against the non-redundant Metazoan database search of conserved domains and the full length sequence showed homologies to RXRs, RARs and ERRs of various species, further suggesting this is an outlier. CgNHR43 DBD grouped with the NR6 subfamily for the ML analysis, but with relatively weak support, and with NR1 for the Bayesian analysis. This was the only *C. gigas* NR that displayed any homology to the NR6 subfamily for either the separate or combined conserved domains sequence. However, the LBD and the DBD plus LBD analyses nested CgNHR43 deeper in the NR1 subfamily. Although CgNH42 and CgNH43 have a classical NR structure and are expressed in oyster tissue there is potential that they are unitary pseudogenes. However, as it is difficult to resolve the nearest common ancestor to these genes, this theory has not been tested and will require future functional assessment.

CgNR1CDEFα and CgNR1CDEFβ associated with the MgNR1DEF and nested with the NR1C, NR1D, NR1E and NR1F groups. The individual conserved domain analyses assigned CgNR1CDEFβ either to the NR1F group (DBD analyses) or NR1E group (ML LBD analysis) than to CgNR1CDEFα or MgNR1DEF. Results of BLASTp search against metazoan protein database found homologies to MgNR1DEF and NR1C-NR1F members of various species for the DBD, LBD and full length sequences.

There were few differences within receptor groups among the phylogenetic trees. The novel receptor group NR1P was highly supported in the DBD plus LBD and the individual LBD phylogenetic analyses (BS = 97-100, PP = 1) and weakly supported in the DBD analysis (BS = 41, PP = 0.81), but the MgNR1G always associated with this group. However, the arrangement of the *C. gigas* receptors within the NR1P group varied marginally between trees. Similar small differences were identified for the four *C. gigas* homologs of the NR2E group. The relationship between all members of this group changed depending on which receptor unit and analysis were used. The DBD analyses showed a few dissimilarities. CgNR1H was placed closer to the human farnesoid X receptor (FXR) compared to the other trees, and revealed higher homologies to the *D. melanogaster* ecdysone receptor EcR; the Hepatocyte Factor 4 (HNF4) homolog CgNR2A is more closely related to the human members of NR2A than to the *Drosophila* homolog; and the molluscan orthologs of NR2B assigned closer to the *Drosophila* ultraspiracle protein USP than to the human RXR homologs.

## Discussion

In this study, 43 nuclear receptors were identified in the bivalve *C. gigas,* representing six of the seven common NR subfamilies. This large set of *C. gigas* NRs provides an overview of nuclear receptor presence in the class Bivalvia and it can enhance the understanding of nuclear receptor evolution in invertebrates and the biological processes in which nuclear receptors are involved. Furthermore, it presents information on possible xenobiotic targets in mollusc species, which occupy an important position in terms of the evolution of the protostomes and as key ecological species in aquatic habitats.

### Novel group NR1P

The phylogenetic analyses of *C. gigas* NRs revealed a novel monophyletic group among the NR1 subfamily including eleven oyster NRs. In addition this group includes one previously characterised *M. galloprovincialis* receptor, MgNR1G. The novel group was supported by high bootstrap values and therefore named as NR1P. The results indicate that the MgNR1G receptor, which was previously assigned to the *C. elegans* Sex-1 NR [22], is not a real homolog to the NR1G group, but a member of the novel NR1P group. Putative functions of these NRs cannot be deduced based on the phylogeny as no close homolog could be identified. However, many members of the NR1 subfamily are involved in growth and development in humans or in moulting and metamorphosis processes in *Drosophila*. A BLASTp search of the conserved domains of NR1P1-NR1P9 against the non-redundant Metazoan database showed weak homologies to NR1C-NR1F invertebrate and vertebrate members, but also relationships to other NR1 groups. CgNR1P10 and CgNR1P11 domains displayed homologies to the NR2E group. These differences in homology are reflected in the different sup-grouping of the NR1P group. However, disagreement in CgNR1P8 and CgNR1P9 positioning among the phylogenetic trees does not allow a separation of NR1P in two separate groups.

The phylogenetic analysis suggests that NR1P segregated from a common ancestor of the NR1C, NR1D, NR1E and NR1F groups. The molluscan phylum separated early among the Protostomia [49] and could have evolved a unique group of NRs. However, it is not clear if this novel group is mollusc specific or also present in other lophotrochozoans. Interestingly, C*. gigas* possesses homologs of NR1C, NR1D, NR1E and NR1F groups, which are also present in Ecdysozoa, the sister clade of the Lophotrochozoa. Therefore, it is possible that some ecdysozoans contain NR1P homologs. However, no NR1P homologs have been identified for *D. melanogaster* (Arthropoda) or *C. elegans* (Nematoda). The nuclear receptor set identified in *Daphnia pulex* (Crustacea) revealed a novel group among the NR1 subfamily, but this group showed high sequence similarities to the invertebrate NR1J group [50]. It is also possible that the ecdysozoans have lost this particular receptor group as has been reported for other NRs [7, 12].

The existence of another novel receptor group in *C. gigas* is implied by the presence of CgNR1CDEFα and CgNR1CDEFβ, which associated with the MgNR1DEF as an outgroup to NR1C-NR1F. However, CgNR1CDEFα and CgNR1CDEFβ were not consistent in their positions for all phylogenetic analyses and therefore, unambiguous assignments are difficult. This inconsistency could be a consequence of rapid evolutionary divergence [22]. It is presumed that the members of the NR1C-NR1F groups originate from a common ancestor, but separated very early in invertebrate evolution [12, 19]. Alternatively, the changing position of CgNR1CDEFβ in the phylogenetic analyses could be a result of sequence similarities. This would be supported by vertebrate RAR-related orphan receptors RORα-γ (NR1F1-3) and REV-ERB receptors, which compete for the same response elements with their DBDs [51]. Additional NR sets of more closely related protostome species are required to confirm the final relationship of these two NRs.

### ***C. gigas*** receptors with functionally characterised homologs

Analysis of the *C. gigas* genome identified a homolog, CgNR1A, to the human thyroid receptors, THRα and THRβ. Vertebrate THRs bind thyroid hormones and play important roles in growth, development and metabolism, and bind either as monomers, homodimers or form heterodimers with RXRs [52]. Though the function of THRs in invertebrates is still unknown, the previously identified flatworm (*S. mansoni*) THRs are able to bind to DNA either as monomers or homodimers and can function as a heterodimer with SmRXR [14, 15].

CgNR1B is a homolog to human RARα-γ paralogs and shows high homology to the freshwater snail *L. stagnalis* RAR [21], with the DBDs and LBDs of these having an amino acid identity of 90% and 73%, respectively. Exposures to all-*trans* retinoid acids (RA) and 9-*cis* RA, known agonistic ligands to vertebrate RAR [53], and to a human RARβ-selective antagonist caused significant deformations to the eyes and shell in *L. stagnalis* embryos [21, 54]. In vertebrates, RARs regulate the expression of genes involved in morphogenesis and especially embryonic development [55].

CgNR1C grouped with the human paralogs of NR1C (PPARα-γ), which bind endogenous ligands, including eicosanoids, fatty acids and fatty acid derivatives. PPARα controls the uptake of fatty acids and their esterification into triglycerides. PPARγ is the main regulator of adipogenesis, fat storage and glucose homeostasis. PPARγ, together with its heterodimer partner RXR, is a potent inducer of adipogenesis in vertebrates when exposed to organotin compounds [56]. PPARβ/δ is involved in fatty acid oxidation, as well as energy consumption and thermogenesis. PPARs are also a target of the fibrate and thiazolidinedione drugs. These are classified as PPARα and PPARγ activators and are used in hyperlipidemia and hyperglycemia treatments [57].

CgNR1D and MgNR1Dv1 group together with the NR1D group human and *Drosophila* homologs. The insect E75 receptor (NR1D3) is induced by ecdysteroids and is involved in moulting and metamorphosis [8, 58]. The human counterparts, REV-ERBα (NR1D1) and REV-ERBβ (NR1D2), display some similar functions to PPARs, playing important roles in lipid and glucose metabolism, gas-response, inflammation and circadian rhythm [59].

The oyster genome also possesses a homolog, CgNR1E, to the *Drosophila* E78 receptor. E78 is directly related to ecdysone signalling and it is another important receptor during metamorphosis [60].

CgNR1F is an ortholog to the *D. melanogaster* DHR3 and *C. elegans* NHR23 receptors. DHR3 is inhibited by E75 and regulates metamorphosis by repressing genes [61]. Expression of the *E75* gene is regulated by another nuclear receptor, ftz-transcription-factor-1, FTZ-F1 (NR5A3) [62]. The human members of the NR1F group, RORα-γ, play a role in circadian rhythm, immune response and other important physiological processes [63].

The oyster genome contains a nuclear receptor (CgNR1H), which is a homolog to the *D. melanogaster* EcR of NR1H group. EcR has also been found in crustaceans [50, 64], in the genome of the mollusc *Lottia gigantea*, leeches and Polychaeta [65]. EcR is involved in moulting, developmental and reproductive processes in insects [8] and crustaceans [64]. In addition, EcR agonists and antagonists are commonly used as insecticides [66]. The vertebrate homologs are liver X receptors (LXRs) and FXR, which regulate lipid and cholesterol metabolism, bile salt synthesis and control expression of certain cytochrome P450s (CYP) [67].

*C. gigas* possesses three NRs of the NR1J group, of which CgNR1Jα and CgNR1Jβ grouped together and CgNR1Jδ assigned on the fringe of the NR1J group. A fourth homolog might be present in the genome as well, indicated by the putative incomplete CgNHR40 NR (Additional file 2), the sequence of which could not be fully resolved. This group appears to be unique to invertebrates, including ecdysozoans [8, 9], crustaceans [50] and platyhelminthes [14]. All *C. gigas* NR1J representatives contained the group-unique residues ESCKAFFR in their DBD sequence [68]. Characterised NR1J receptors include DHR96, which is believed to play a role in xenobiotic stress response [69] and is able to bind cholesterol to regulate cholesterol homeostasis [70]. Xenobiotic defence in *C. elegans* is thought to be managed by the NHR-8 [71]. DAF-12, also a *C. elegans* NR1J, is involved in dauer formation, in which larval development is diverted under adverse environmental conditions to a form of stasis termed the dauer stage [68]. The NR1I subgroup is the vertebrate group homologous to NR1J and shares common receptor ancestors prior to the divergence of deuterostomes and protostomes [12]. Its three representatives, pregnane X receptor (PXR), constitutive androstane receptor (CAR) and vitamin D receptor (VDR), have all been implicated in the vertebrate response to xenobiotic stress [72].

A single member, CgNR2A, of the NR2A (HNF4) group has been identified in the *C. gigas* genome. The NR2A group contains the most ancient NRs found in animals and have been discovered in simple metazoans [4, 5, 7, 73]. Only a single ortholog is encoded in *D. melanogaster*. It is involved in the development of the digestive tract [74], lipid metabolism and mobilization [75]. In humans, HNF4 receptors play a significant role in diseases like diabetes [76] and colon cancer [77].

The *C. gigas* RXR ortholog (CgNR2B) clustered together with other identified molluscan RXRs and the conserved regions had identities of over 93% to the retinoid X receptors’ DBDs and LBDs of *Biomphalaria glabrata* and *L. stagnalis*. The *B. glabrata* RXR is able to act as a heterodimer partner to human NRs and is also able to form homodimers [78]. Retinoid acid (9-cis RA) and docosahexaenoic acid (DHA), natural ligands of vertebrate RXRs [79], have been identified as putative gastropod agonistic ligands [78], while vertebrate RXR pan-antagonists successfully inhibited growth cone turning in adult gastropod CNS and produced eye and shell deformation in embryos during the gastrulation stage [80].

The *C. gigas* receptor CgNR2CD could not be unambiguously assigned to either the NR2C or NR2D group. In humans the NR2C proteins, TR2 and TR4, act as transcriptional repressors in cooperation with co-factors [81]. The *Drosophila* NR2D ortholog DHR78 uses a similar repression mechanism (binding-site competition) and inhibits ecdysone signalling [82]. The *C. elegans* homolog NHR41 is also involved in moulting processes and morphogenesis [83].

CgNR2E1, CgNR2E2, CgN2E3 and CgNR2E5 represent four putative nuclear receptors from the NR2E group. CgNR2E1 and CgNR2E2 have most identity to the *Drosophila* homologs tailless (DmTLL), dissatisfaction (DmDSF) and human TLX receptors. They are all involved in anterior-posterior axis formation and have important roles in vision and forebrain development [8], as well as in emotional behaviour [84]. Furthermore, TLX regulates adult vertebrate neural stem cell proliferation [85]. CgNR2E3 shows homology to vertebrate photoreceptor cell-specific nuclear receptor (PNR), *C. elegans* Fax-1 and *Drosophila* DHR51. PNR is required for controlling neural differentiation and retina development [86, 87] and neuron identity in *C. elegans* is regulated by FAX-1 [88]. CgNR2E5 shows homologies to DHR83, a *Drosophila* function-unknown receptor [8].

The *C. gigas* genome possesses one homolog (CgNR2F) to the NR2F group, which has a close phylogenetic relationship to the *D. melanogaster* seven-up receptor, DmSVP. Similar to other members of NR2E the DmSVP and HsCOUP-TF1/2 receptors are responsible for neural development and photoreceptor cells [89–92].

Two NR3 homologs were identified in the *C. gigas* genome. CgNR3A, previously identified as CgER, grouped well with other molluscan ERs and has been shown to be unresponsive to estrogen [93]. The second, CgNR3B is a member of the NR3B group, representing the ‘constitutively activated’ ERRs, and shows a high degree of similarity to the previously identified *M. cornuarietis* molluscan ERR (DBD = 94.6% and LBD = 65.6%, respectively). McERR has been tested for modulation by vertebrate estrogens and other putative ligands *in vitro* and *in vivo*. However, no significant evidence for modulation could be identified and it is assumed that McERR is ‘constitutively activated’ [20, 94]. CgNR3B can be assumed to work in a similar way of action regarding ligand binding and activation, particularly as ligand-activated-requirement for a NR3B representative has not been identified either in invertebrates or vertebrates. No further NR3 subfamily receptor were identified by the genome analysis of *C. gigas*, which is consistent with the theory of the NR3 subfamily evolution [12, 50, 95–97] that the expansion of steroid receptors including the deuterostome specific NR3C group occurred after the divergence of protostomes and deuterostomes. However, we cannot rule out that additional steroid receptors exist in protostomes, or even in other mollusc species, and have then been lost during gene deletion events [98].

The CgNR4A receptor is the sole *C. gigas* member of the NR4 subfamily. There might be another homolog, CgNHR41, but this NR contains only a single DBD; an LBD could not be identified (Additional file 2). The human NR4 subfamily includes nerve growth factor I-B (NGFI-B), nuclear receptor related 1 protein (NURR1), neuron derived orphan receptor 1 (NOR1), and is involved in a broad array of cellular metabolic processes; vascular remodelling and cancer [99, 100]. In *Drosophila* species the NR4 gene DHR38 mediates an ecdysteroid signalling pathway [101] and the *C. elegans* homolog NHR-6 is involved in ovulation processes [83]. The LBDs of NR4 genes found in humans, *D. melanogaster* and *S. mansoni* contain phenylalanines, which fill the entire volume of the ligand binding pockets and NR4 subfamily members are therefore suggested to be “true orphans” requiring no ligand [101–104]. CgNR4A contains phenylalanine at the same positions in its LBD, suggesting it too is a “true orphan” receptor.

The NR5 group is represented by two nuclear receptor homologs in *C. gigas*, designated CgNR5A and CgNR5B. The CgNR5A DBD contains a highly conserved sequence (FTZ-F1 box), which is characteristic of the NR5A group. This sequence is located at the boundary between the DBD and the hinge region and is essential for the high-affinity interaction with the DNA [105]. The *D. melanogaster* FTZ-F1 receptor is part of the ecdysteroid regulated nuclear receptor group including members of different NRs subfamilies. Expression profiles of various fly NRs showed the close relationship between EcR, E75, E78, DHR3, DHR4, FTZ-F1 and DHR39 [8, 106] and as a part of this regulatory cascade FTZ-F1 has a crucial role during embryonic development and metamorphosis [8, 106]. The *C. elegans* NHR25 receptor is also associated with reproduction, embryogenesis and moulting processes in Nematodes [68]. The human NR5A genes include liver receptor homolog-1 (LRH1), which regulates bile acid and cholesterol metabolism [107], and the steroidogenic factor 1 (SF1), which is involved in reproductive development and endocrine function [108]. CgNR5B is a member of the NR5B group represented by the *D. melanogaster* DHR39 receptor. Besides its role in embryonic development and metamorphism DHR39 is also involved in female reproductive tract development [109]. Homologs of this group are also identified in crustaceans [50] and the invertebrate flatworm *S. mansoni*[110]. *D. melanogaster* DHR39 and FTZ-F1 receptors also provide a good example that nuclear receptors are able to compete for the same DNA binding site as a putative target-gene regulated mechanism [111, 8].

It is worth noting that *C. gigas* does not contain a NR6 subfamily homolog. Homologs have been identified both in protostomes (ecdysozoans e.g. *D. melanogaster* DHR4 [106], *C. elegans* NHR91 [9] and Crustacea [50]) and deuterostomes (germ cell nuclear factor (GCNF) in *H. sapiens*). Thus, it is likely that the NR6 subfamily homolog in the Pacific oyster could have been lost due to a gene loss event either during the separation of ecdyzoans and lophotrochozoans or during one of the evolutionary differentiations to the Pacific oyster.

CgNR0B is a predicted member of the miscellaneous NR-subfamily NR0B, lacking the DNA binding domain. The first human member of NR0B, DAX-1, plays major roles in steroidogenesis and reproductive development [112] and acts as a dominant-negative regulator of other NR transcription, e.g. SF1 and ER [113]. SHP, the second human NR0B representative, is involved in maintaining cholesterol and glucose homeostasis [112].

Two nuclear receptors, Cg2DBDγ and Cg2DBDδ, associate with the 2DBDNR group found in *S. mansoni*[14]. Cg2DBDγ and the *S. mansoni* receptors each contain the same unique P-box sequences, CEACKK, in the first DBD sequence [14, 15]. The two DBDs and the LBD sequence show amino acid identities of 52% and 25% respectively, to the Sm2DBDγ. Cg2DBDδ, which also possesses two DBDs, does not assign as closely to Sm2DBD receptors as Cg2DBDγ for its second DBD and LBD. In addition, it does not contain the unique P-box sequence in its first DBD. However, it contains another unique P-box sequence, CLPCKS, which has not been identified in any other nuclear receptor’s DBD. It seems that this 2DBD receptor may be unique to molluscs. Further phylogenetic analyses of NRs in other species and functional studies of Cg2DBDδ will reveal if this is a functional molluscan specific 2DBD receptor.

### Molluscan nuclear receptors as xenobiotic targets

The variety of NRs in *C. gigas* and the known propensity for NRs to bind ligands prolifically provides the opportunity for xenobiotic disruption and chemically induced biological effects. Exposure to TBT can cause multiple developmental problems in *C. gigas*, including shell thickening [33–36]. TBT, a ligand for vertebrate RXR/PPAR heterodimers [114, 115], and thought to interact with molluscan RXRs [29, 37–47] may also target the *C. gigas* RXR homolog. Consequently, disruption of RXR function, the putative exclusive heterodimer partner of NRs, could cause alteration of gene regulation and the reported malformations. Additionally, CgNR1C, the homolog to the human PPARs, could be affected by TBT. Gastropods exposed to rosiglitazone, a known vertebrate PPAR ligand, exhibited similar effects (imposex) to TBT exposed animals [47]. A PPAR homolog in gastropods has not yet been identified, but it is likely that gastropod species also contain a PPAR homolog due to the high conservation of similar nuclear receptor complements between related species [12].

Furthermore, the *C. gigas* PPAR homolog could be affected by environmental xenobiotics in addition to TBT. Peroxisome proliferation in bivalve species is actually used as a biomarker for monitoring the health of aquatic environments [116]. Organic xenobiotics such as polycyclic aromatic hydrocarbons (PAHs), phthalates and bisphenol-A, increase the number and volume of peroxisomes and induce peroxisomal β-oxidation enzyme acyl coenzyme A (acyl-CoA) oxidase in *Mytilus edulis* and *M. galloprovincialis*[117–119]. Vertebrate peroxisome proliferation and acyl-CoA are regulated by PPARs [120, 121] and disturbance of PPAR regulated genes has been observed after exposure to the aforementioned xenobiotics [122–124].

The effect of xenobiotic vertebrate sex steroids is also a widely debated topic and has been investigated in a large range of mollusc species. Several studies have reported effects on reproductive output and morphology in different molluscan classes when exposed to vertebrate estrogen E2, synthetic estrogens and estrogen mimics [125], but their response remains ambiguous and characterised largely through hypothesis and homology. It was assumed that estrogen and other sex steroids (androgens, progestins, and corticoids) are used as reproductive hormones, operating through steroid receptors of the NR3 subfamily [126–129] and possessing a vertebrate-like sex steroid biosynthetic pathway [130, 131]. However, functional studies have shown that neither molluscan ERs nor ERR bind to estrogens or other sex steroids [19, 20, 93, 94, 132–134] and ER and ERR gene transcription was not affected by exogenous estrogens [94, 135]. This present study identified two NR3 members, including an ER homolog, that does not bind estrogen [93] and an ERR homolog, which is unlikely to bind estrogen. Additional NR3 members, which could interact with vertebrate sex steroids, could not be identified. This supports the hypothesis that any mechanism of sex steroid actions in molluscs does not operate in a similar way to those in vertebrates and is not mediated via the NR3 group of nuclear receptors [125, 136]. However, our findings do not provide any new information for the debate on the ligand state of the putative ancestral steroid receptor before the deuterostomes and protostomes have separated, which hypothesizes either a ligand-regulation, a sensory function or a constitutive action [96, 97].

Nevertheless, estrogens and sex steroids still might have xenobiotic effects on mollusc species possibly mediated via alternative NRs. For example, *C. gigas* possesses three NR1J homologs, which are known to respond to estrogens in *D. pulex* (DHR96) [137]. Similar results exist for vertebrate NR1I members, PXR and CAR [138, 139].

## Conclusion

This study verified the presence of 43 NRs in the Pacific oyster, *C. gigas.* Phylogenetic analyses demonstrate that the majority of *C. gigas* NRs are homologs to *D. melanogaster* and human NRs supporting the theory that these receptor groups emerged prior to the divergence of the Bilateria [7, 12]. A novel group, NR1P, was discovered in *C. gigas*, which could not be identified in ecdysozoans or humans. Further studies of NRs in closely related mollusc species and in non-molluscan lophotrochozoans will discover if this novel group is mollusc specific or also present in other lophotrochozoan phyla. The *C. gigas* NR family does not contain any additional homolog to NR3 groups beside the ER and ERR and therefore, supports the theory that steroid ligand expansion of sex steroid NR3 subfamily is deuterostome specific.

*C. gigas* is a key ecological species and an important food source for humans, but due to its filter feeding lifestyle, it is susceptible to environmental pollution. This set of NRs provides important information on putative xenobiotic targets and the discovery of PPAR and RXR homologs in *C. gigas* encourages the theory of an RXR/PPAR heterodimer involvement in effects caused by TBT contamination. Additionally, we found further evidence that exogenous estrogens do not operate through a NR3 subfamily member, simply by the absence of an adequate NR3 candidate for estrogen binding.

The *C. gigas* NRs provide a valuable illustration of the presence and importance of this superfamily of ligand-regulated transcription factors, leaving the way open for future studies to analyse their functional significance.

## Methods

### Identification of nuclear receptors in ***C. gigas*** genome

Putative *C. gigas* NR sequences were identified through a local combination of tBLASTn and BLASTp searches of genome, CDS and protein databases using the published Pacific oyster genome [48]. The protein sequences (full length sequences, isolated DBD regions and isolated LBD regions) of the 48 *H. sapiens* and 21 *D. melanogaster* NRs were downloaded from GenBank and were used as templates for interrogating the *C. gigas* databases. The DBD (zf-C4) and LBD (hormone_rec) of identified putative oyster NRs were verified by using Pfam (Pfam 26.0) [140] and in addition annotated by using the Conserved Domain Database at NCBI [141]. A BLASTp search using the conserved domains and the full length sequences against the non-redundant (nr) Metazoan protein database at NCBI was used for a first characterization of the putative nuclear receptors.

Nomenclature of the putative *C. gigas* nuclear receptors was based on phylogenetic analyses using conserved domains and sequence similarities of full length sequences to the NRs from *H. sapiens* and *D. melanogaster*. Genes were classified to nuclear receptor subfamily groups based on the nomenclature guidelines [13], if a single representative was identified. For groups, which include several representatives, the nomenclature name of the closest orthologs were given or listed with the Greek suffix α-δ. Nuclear receptors, which showed similarities to two or more NR subfamily groups, were named after all group names. Nuclear receptors, which could not be assigned to a NR subfamily group or for which sequence could not be resolved, were labelled as CgNHRs.

### Verification ***C. gigas*** nuclear receptor expression

Six *C. gigas* individuals were sampled from the coastline close to Starcross, Devon, UK (50.6167° N, 3.4500° W). Shucked whole animals were frozen in liquid nitrogen and ground to a fine powder. Total RNA was extracted from this homogenate using TRI Reagent RNA Isolation Reagent (Sigma-Aldrich) following the manufacturer’s protocol [142] and DNA removed with RQ1 RNase-Free DNase (Promega). RNA was cleaned using the RNeasy Mini Kit (QIAGEN) and pooled. Cleaned total RNA was converted to cDNA with the ThermoScript RT/PCR System (Invitrogen) using oligo(dT) primers. Forward and reverse primers for 42 putative NRs were designed with Primer-Blast at National Centre for Biotechnology Information (NCBI) [143] to amplify either parts of the hinge domain plus LBD, or parts of LBD or whole DBDs with predicted amplicons of 177-984 bp (Additional file 4). Primers for CgNR1D were also designed to obtain the NR sequence. One microlitre of undiluted cDNA was used for PCR amplification with the GoTaq system (Promega) under the following conditions: 95°C 5 min, thirty-five cycles of 95°C for 15 s, 57°C for 30 s, 72°C for 1.5 min, and a final extension at 72°C for 5 min. Amplified PCR products were visualized on a 1.5% agarose gel and amplicons purified with the QIAquick PCR Purification Kit, or with QIAquick Gel Extraction Kit (Qiagen, UK) and sequence verification conducted by Eurofins MWG Operon (Ebersberg, Germany).

### Phylogenetic analysis

Thirty-eight classically and five abnormally structured putative NRs from *C. gigas* were compared to 48 *H. sapiens*, 21 *D. melanogaster*, 12 *C. elegans*, 3 *S. mansoni* and 16 previously cloned nuclear receptor amino acid sequences from different mollusc species (*A. californica, B. glabrata, C. gigas, L. stagnalis, M. cornuarietis, M. edulis, M. galloprovincialis, Nucella lapillus, Octopus vulgaris, Thais clavigera*). The lineage-specific expansion of *C. elegans* in the NR2A subfamily was disregarded as preliminary data of *C. gigas* NRs did not suggest a similar NR2A expansion. For a better readability of the phylogenetic trees only two NRs, representatives of the *C. elegans* NR2A subfamily, have been used. The amino acid sequence GenBank accession numbers of all nuclear receptor used in the phylogenetic analysis is available in Additional file 1. The DBD and LBD amino acid sequences were aligned using default parameters in MUSCLE v3.8.31 [144] and edited manually in case of errors. LBD domains were trimmed to allow efficient alignment of conserved sequences. Three separate maximum likelihood (ML) analyses were conducted, the first using only the DBD, the second with a portion of the LBD, and the third with the DBD plus a portion of the LBD. Trees were constructed using PhyML v3.0 [145] with a LG + I + G matrix (model determined by AIC criteria with ProtTest v2.4) [146]. Nodes were supported by ML analyses assessed with 1,000 bootstraps. The same three data sets were also tested by Bayesian Inference, carried out under a proportion of invariable sites and gamma-distributed rate heterogeneity among sites with a mixed amino acid replacement model using MrBAYES v3.2.2 [147]. The trees started randomly with four simultaneous Markov chains running for 5 million generations with chains sampled every 100 generations and with a burnin of 5000 trees. The JTT model [148] was selected as the best fitting substitution model. The Bayesian posterior probabilities (PPs) were calculated using a Markov chain Monte Carlo (MCMC) sampling approach implemented in MrBAYES v3.2.2. Additional phylogenetic support was conducted by a distance neighbor-joining (NJ) analysis of the DBD plus the portion of LBD using Seaview v4.0 [149]. Default characteristics were used and the branch support was measured by bootstrap analysis with 1,000 replicates. Phylogenetic trees were visualized and illustrated with FigTree v1.4.0 (http://tree.bio.ed.ac.uk/software/figtree/). Phylogenetic data has been uploaded to TreeBASE where it is available for public access via the website and study ID 15636 (http://purl.org/phylo/treebase/phylows/study/TB2:S15636).

## Electronic supplementary material

Additional file 1: **Nuclear receptor amino acid sequence GenBank accession numbers for** ***Crassostrea gigas,*** ***Homo sapiens, Drosophila melanogaster, Caenorhabditis elegans*** **and mollusc species **[19–22, 37, 39, 44, 78, 80, 93, 132, 135, 150–153]. (XLS 40 KB)

Additional file 2: **Phylogenetic tree using only DBD of NR alignment conducted by a Maximum likelihood (ML) and Bayesian Inference analyses.** ML bootstrap support values (percentage of 1000 BS) and Bayesian posterior probabilities (PPs) are provided above the nodes separated by slash. Star indicates the node obtained from the Bayesian Inference analysis, which was different from that obtained by ML method. *Crassostrea gigas* NRs highlighted in red. Ac: *Aplysia californica,* Bg: *Biomphalaria glabrata,* Ce: *Caenorhabditis elegans*, Cg: *C. gigas,* Dm: *Drosophila melanogaster*, Hs: *Homo sapiens*, Lym: *Lymnea stagnalis*, Mc: *Marisa cornuarietis*, Me: *Mytilus edulis*, Mg: *Mytilus galloprovincialis*, Nl: *Nucella lapillus,* Ov: *Octopus vulgaris,* Sm: *Schistosoma mansoni,* Tc: *Thais clavigera.* (PDF 396 KB)

Additional file 3: **Phylogenetic tree using only a portion of LBD of NR alignment conducted by a Maximum likelihood (ML) and Bayesian Inference analyses.** ML bootstrap support values (percentage of 1000 BS) and Bayesian posterior probabilities (PPs) are provided above the nodes separated by slash. Star indicates the node obtained from the Bayesian Inference analysis, which was different from that obtained by ML method. *Crassostrea gigas* NRs highlighted in red. Ac: *Aplysia californica,* Bg: *Biomphalaria glabrata,* Ce: *Caenorhabditis elegans*, Cg: *C. gigas,* Dm: *Drosophila melanogaster*, Hs: *Homo sapiens*, Lym: *Lymnea stagnalis*, Mc: *Marisa cornuarietis*, Me: *Mytilus edulis*, Mg: *Mytilus galloprovincialis*, Nl: *Nucella lapillus,* Ov: *Octopus vulgaris,* Sm: *Schistosoma mansoni,* Tc: *Thais clavigera.* (PDF 393 KB)

Additional file 4: **Table of primer sequences including amplicon length (bp) and parts of nuclear receptor domains sequenced: A/B = N-terminal domain, C = DNA binding domain, D = hinge domain, E = ligand binding domain, F = C-terminal domain.** (XLS 26 KB)
