# Supplementary figures and images for: The nuclear receptor gene family in the Pacific oyster, Crassostrea gigas, contains a novel subfamily group
Source: BMC Genomics. 2014 May 15;15:369. doi: 10.1186/1471-2164-15-369 (PMC4070562; doi:10.1186/1471-2164-15-369)

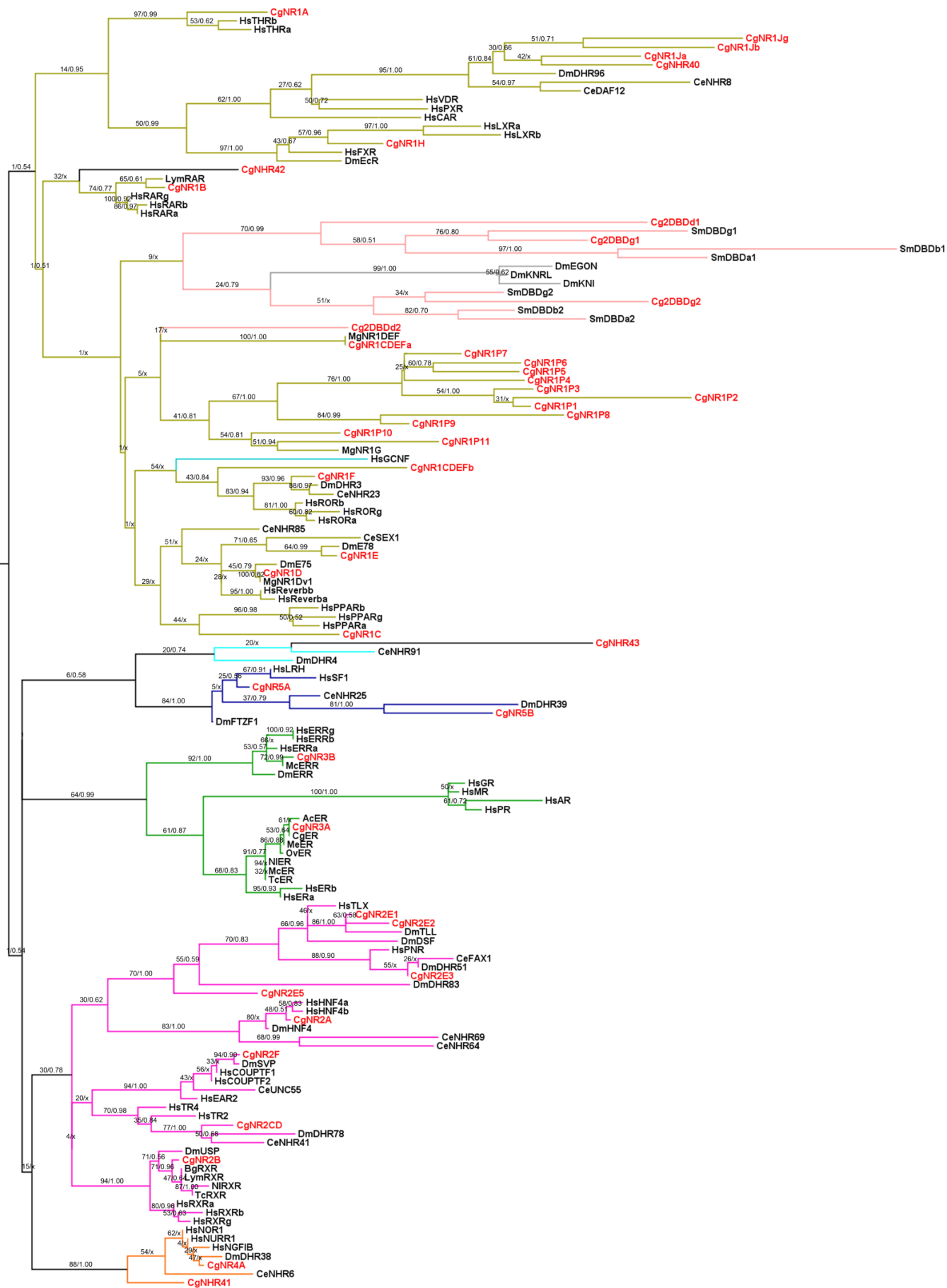

Supplement: Supplementary file 2 — Additional file 2: Phylogenetic tree using only DBD of NR alignment conducted by a Maximum likelihood (ML) and Bayesian Inference analyses. ML bootstrap support values (percentage of 1000 BS) and Bayesian posterior probabilities (PPs) are provided above the nodes separated by slash. Star indicates the node obtained from the Bayesian Inference analysis, which was different from that obtained by ML method. Crassostrea gigas NRs highlighted in red. Ac: Aplysia californica, Bg: Biomphalaria glabrata, Ce: Caenorhabditis elegans, Cg: C. gigas, Dm: Drosophila melanogaster, Hs: Homo sapiens, Lym: Lymnea stagnalis, Mc: Marisa cornuarietis, Me: Mytilus edulis, Mg: Mytilus galloprovincialis, Nl: Nucella lapillus, Ov: Octopus vulgaris, Sm: Schistosoma mansoni, Tc: Thais clavigera. (PDF 396 KB) [file 12864_2013_6129_MOESM2_ESM.pdf]

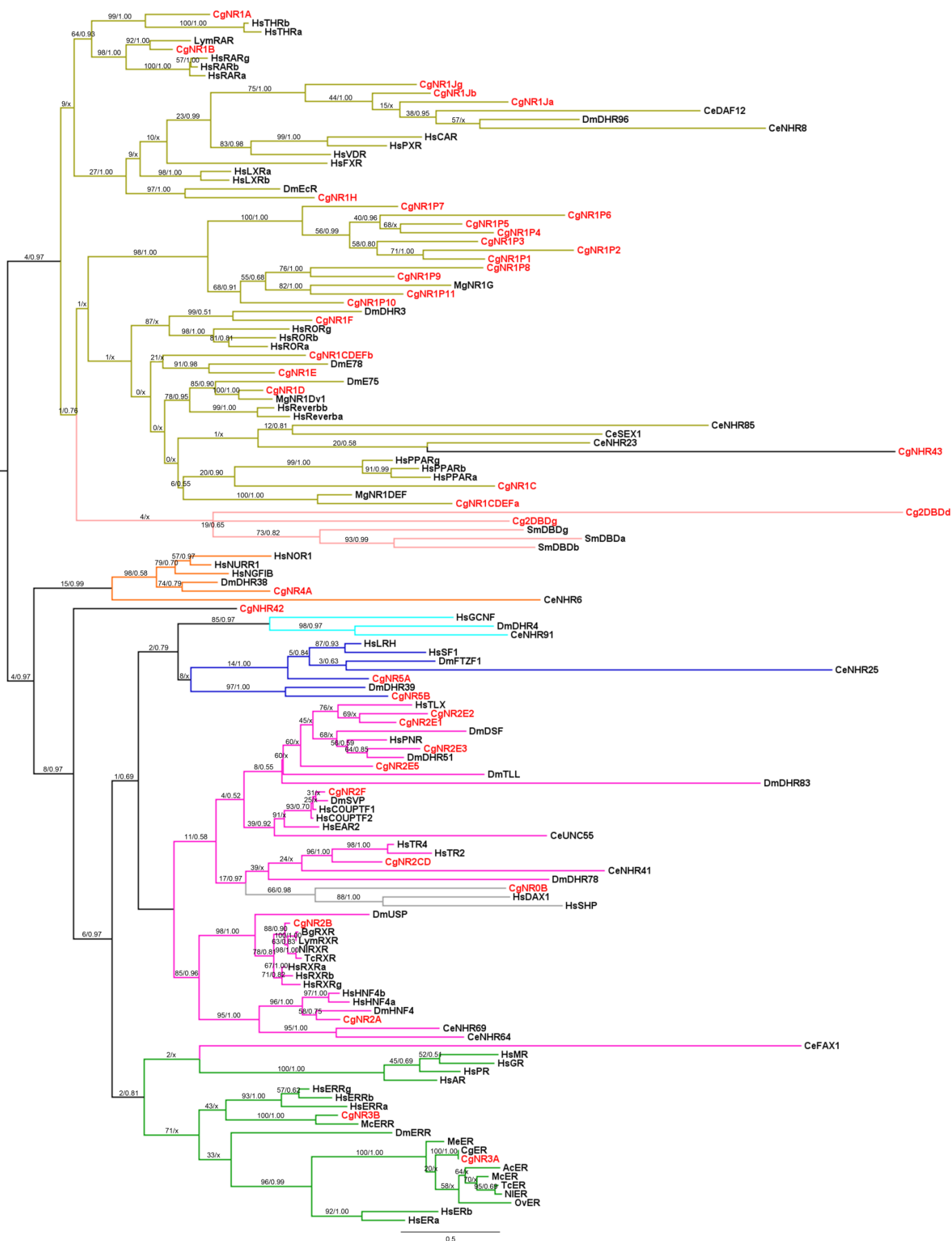

Supplement: Supplementary file 3 — Additional file 3: Phylogenetic tree using only a portion of LBD of NR alignment conducted by a Maximum likelihood (ML) and Bayesian Inference analyses. ML bootstrap support values (percentage of 1000 BS) and Bayesian posterior probabilities (PPs) are provided above the nodes separated by slash. Star indicates the node obtained from the Bayesian Inference analysis, which was different from that obtained by ML method. Crassostrea gigas NRs highlighted in red. Ac: Aplysia californica, Bg: Biomphalaria glabrata, Ce: Caenorhabditis elegans, Cg: C. gigas, Dm: Drosophila melanogaster, Hs: Homo sapiens, Lym: Lymnea stagnalis, Mc: Marisa cornuarietis, Me: Mytilus edulis, Mg: Mytilus galloprovincialis, Nl: Nucella lapillus, Ov: Octopus vulgaris, Sm: Schistosoma mansoni, Tc: Thais clavigera. (PDF 393 KB) [file 12864_2013_6129_MOESM3_ESM.pdf]
